# A spatio-temporal autoregressive model for monitoring and predicting COVID infection rates

**DOI:** 10.1007/s10109-021-00366-2

**Published:** 2022-04-26

**Authors:** Peter Congdon

**Affiliations:** School of Geography, Queen Mary University of London, Mile End Rd, London, E1 4NS UK

**Keywords:** Autoregressive, Epidemic, Clustering, Forecasting, Spatio-temporal, Bayesian, COVID-19, C23, C11, C32

## Abstract

The COVID-19 epidemic has raised major issues with regard to modelling and forecasting outcomes such as cases, deaths and hospitalisations. In particular, the forecasting of area-specific counts of infectious disease poses problems when counts are changing rapidly and there are infection hotspots, as in epidemic situations. Such forecasts are of central importance for prioritizing interventions or making severity designations for different areas. In this paper, we consider different specifications of autoregressive dependence in incidence counts as these may considerably impact on adaptivity in epidemic situations. In particular, we introduce parameters to allow temporal adaptivity in autoregressive dependence. A case study considers COVID-19 data for 144 English local authorities during the UK epidemic second wave in late 2020 and early 2021, which demonstrate geographical clustering in new cases—linked to the then emergent alpha variant. The model allows for both spatial and time variation in autoregressive effects. We assess sensitivity in short-term predictions and fit to specification (spatial vs space-time autoregression, linear vs log-linear, and form of space decay), and show improved one-step ahead and in-sample prediction using space-time autoregression including temporal adaptivity.

## Introduction

Forecasts of future infectious disease incidence have had major policy importance, for example in the COVID-19 epidemic of 2020-2021. However, even short-term forecasts may face difficulties in practice. These include limited data, quantifying forecast uncertainty, and specification issues (Petropoulos and Makridakis 2020; Roda et al. 2020; Stehlík et al. 2020). Where separate infection time series for a number of areas are available, this may assist forecasts through a borrowing strength mechanism (Haining et al. 2021), with Shand et al. (2018) noting the gain from taking “advantage of the spatial and temporal dependence structures so that the statistical inference at one location can borrow strength from neighbouring regions in both space and time”. However, modelling and predicting area trajectories in infectious disease poses particular problems when counts are changing rapidly in epidemic situations, and there may well be geographic infection hotspots.

Notions of borrowing strength through spatial random effects are a major feature of the Bayesian disease mapping approach for area disease counts (Kang et al. 2016), and adaptations of disease mapping to modelling longitudinal infectious disease data have been discussed in a number of papers (e.g. Clements et al. 2006; Coly et al. 2021). Consider, in particular, applications to epidemic time series for sets of administrative areas, which are available in several countries for the COVID-19 epidemic. A widely adopted strategy for such data, aiming at short term prediction, involves low order autoregression in infectious disease counts or rates, in both an area itself (the focus area), and in areas surrounding the focus area (Shand et al. 2018; Paul and Held 2011). Existing approaches have focussed on spatial variation in autoregressive dependence, so allowing for geographic heterogeneity (Dowdy et al. 2012).

The contribution and novelty of this paper is to show how different specifications of autoregressive dependence in incidence counts may considerably impact on adaptivity in epidemic situations. In particular, we introduce temporal as well as spatial variation in autoregressive dependence and show that this feature provides much improved predictive performance in situations where infection counts are rapidly changing.

Such rapid fluctuations in cases, associated with multiple epidemic waves, have been a feature of the COVID-19 epidemic. Sharp upward trends in cases have initially tended to be geographically concentrated, with subsequent diffusion away from initial hotspots (Dowdy et al. 2012). Effective policy responses in such situations depend on forecasting approaches that provide a perspective on short-term future implications of current trends (Shinde et al. 2020). In particular, geographically disaggregated forecasts are important for prioritizing interventions or severity designations, such as the “local tiers” in the UK COVID-19 policy response (Hunter et al. 2021).

The approach used here can potentially be generalized to model longitudinal count data in non-disease applications involving areas, or for longitudinal count data for units other than areas. An example of the former might be applications involving spatial forecasting and spatial diffusion of count data (e.g. Glaser 2017; Glaser et al. 2021). Examples of such diffusion include behavioural copycat effects (Schweikert et al. 2021).

In this paper, we assess predictive performance of an autoregressive model for infectious disease counts, applied to COVID-19 data for 144 English local authorities during the UK epidemic second wave—at the end of 2020 and into early 2021. These local authorities are in the South East of England, where a sharp (and geographically concentrated) upturn in cases in late 2020 was attributed to the emergence of a new COVID variant, the “Kent variant” or alpha variant (World Health Organization 2021). The model proposed here allows for both spatial and time variation in autoregression coefficients. We show clear gains in prediction over a less general specification. Impacts of alternative model features are considered, namely the choice between a linear (identity link) or log-linear model form, and the assumed form of weighting infections in neighbouring areas. We use Bayesian inference and estimation, via the BUGS (Bayesian inference Using Gibbs Sampling) package (Lunn et al. 2009).

## Related Research

The typical form of data encountered in analysis of spatio-temporal infections data involves incidence counts $y_{it}$ for areas $i=1,...,N$ and times $t=1,...,T$. However, some spatio-temporal models for such data have used normalizing transformations of originally count data. Thus, Shand et al. (2018) consider a logarithmic transformation of yearly HIV diagnosis rates (per 100,000 population) for US counties.

Alternatively for models applied specifically to counts, Poisson and negative binomial time series regression methods may be used. Other count distributions may be used (Jalilian and Mateu 2021; Yu 2020). Spatio-temporal adaptations of disease mapping have been applied to analysis of infections, including across and within area random walks (e.g. Zhang et al. 2019; Jalilian and Mateu 2021; Lowe et al. 2021). Both Shand et al. (2018) and Paul and Held (2011), use spatially varying auto-regression applied either to lagged infection counts in an area itself (the focus area), or to areas surrounding the focus area (the neighbourhood), or both. A geographically adaptive scheme is also used by Lawson and Song (2010) in analysis of foot and mouth disease data. Lawson and Song (2010) use a focus area and neighbourhood lag in flu infection counts as an offset (with known coefficient) in Poisson regression, with an application to COVID forecasts by area in Sartorius et al. (2021). Applications to COVID-19 forecasting, based on Paul and Held (2011), are provided by Giuliani et al. (2020) and Rui et al. (2021). Detection of space-time clusters in COVID-19 is exemplified by Martines et al. (2021).

For applications without spatial disaggregation, a wide range of methods have been used for COVID-19, and infectious diseases generally. These include autoregressive integrated moving average (ARIMA) models (e.g. Maleki et al. 2020; Chintalapudi et al. 2020; Petukhova et al. 2018), integer-valued autoregressive (INAR) models (Chattopadhyay et al. 2021), exponential smoothing (Petropoulos and Makridakis (2020); Gecili et al. (2021)), or bivariate forecasts. For example, the study by Johndrow et al. (2020) models COVID deaths as a lagged function of earlier new cases. For infectious diseases with an established seasonal pattern, SARIMA (seasonal ARIMA) forecasting has been used (Qiu et al. 2021). Applications of phenomenological models to COVID-19 incidence forecasts—based on mathematical representations of epidemic curves, such as the Richards model (Richards 1959)—include Roosa et al. (2020).

## Methods

We focus here on infectious disease models using count data regression. We consider first models for count time series, without area disaggregation, as these can provide a basis for generalisation to area-time data. Relevant specifications may specify AR dependence on previous counts, or on previous latent means; models with autoregressive (AR) dependent errors (Hay and Pettitt 2001) may also be considered (Jalilian and Mateu 2021).

### Time dependent autoregressive count data models

Consider Poisson distributed counts at times $t=1,...,T,$ namely $y_{t}$ $\thicksim Poi(\mu _{t}),$ (with *Poi* for Poisson density, with means $\mu _{t}),$ or negative binomial (NB) counts, $y_{t}$ $\thicksim Negbin(\mu _{t},\Omega )$ (with *Negbin* for negative binomial density, with means $\mu _{t}$ and dispersion parameter $\Omega )$. The parameterisation of the negative binomial is as in Zhou et al. (2012), namely

$$\begin{aligned} p(y|\mu ,\Omega )=\frac{(y+\Omega -1)!}{y!(\Omega -1)!}\left( \frac{\mu }{ \mu +\Omega }\right) ^{y}\left( \frac{\Omega }{\mu +\Omega }\right) ^{\Omega }. \end{aligned}$$

In a simple autoregressive representation (Fokianos 2011), one may adopt an identity link, and, subject to suitable parameter constraints, specify AR1 (AR with first-order lag) dependence in lagged counts $y_{t-1}$ and in latent means $\mu _{t-1}.$ The general form of this representation is termed the autoregressive conditional Poisson (ACP) model by Heinen (2003), or the linear model by Fokianos (2011). Thus

1 $$\begin{aligned} \mu _{t}=\phi +\alpha y_{t-1}+\gamma \mu _{t-1}, \end{aligned}$$

where $\phi$, $\alpha ,$ and $\gamma$ are all positive. An alternative log-linear model (Fokianos and Tjøstheim 2011) has a log-link with

2 $$\begin{aligned} \log (\mu _{t})=\nu _{t}=f+a\log (y_{t-1}+1)+c\nu _{t-1}, \end{aligned}$$

where $\nu _{t}$ and $\nu _{t-1}$ are the logarithms of $\mu _{t}$ and $\mu _{t-1}$ respectively,  *f* is an intercept, and *a* and *c* are autoregressive coefficients.

In both Eqs. (1) and (2), the autoregressive coefficients could be taken as time varying, namely $\{\alpha _{t},\gamma _{t}\}$ and $\{a_{t},c_{t}\}.$ Varying intercepts to represent time dependent effects other than autoregressive, could also be added. For example in Eq. (1), one may take

$$\begin{aligned} \phi _{t}=\exp (\phi _{0}+\eta _{t}), \end{aligned}$$

where $\eta _{t}\thicksim \mathcal {N}(\eta _{t-1},\sigma _{\eta }^{2})$ is a random walk with variance $\sigma _{\eta }^{2}$. However, random coefficients research so far have concentrated on random coefficient AR models, without lags in latent means (e.g. Sáfadi and Morettin 2003).

### Random coefficient autoregressive area-time models

To generalize these representations to area-time infection count data (areas $$i=1,...,N$$), one may add lags to infection counts in spatially close areas (Martines et al. 2021). These reflect geographic infection spillover—due, for example, to social interactions between residents in different areas, or to cross boundary commuting (Mitze and Kosfeld 2021). To allow for spatial lag effects, let $$w_{ij}$$ be row standardised spatial weights expressing spatial interaction between areas *i* and *j*, with $$\underset{j}{\sum } w_{ij}=1$$. They may be based on adjacency of areas, or distances between them. For example, let $$h_{ij}=1$$ for adjacent areas (with $$h_{ii}=0$$), and $$ h_{ij}=0$$ otherwise. Then, $$w_{ij}=h_{ij}/\underset{j}{\sum }h_{ij}.$$ Consider Poisson distributed counts $$y_{it}$$ $$\thicksim Poi(\mu _{it}),$$ or NB counts, $$y_{it}$$ $$\thicksim Negbin(\mu _{it},\Psi ).$$

As in panel data analysis (Greene 2011), randomly varying autoregressive parameters can be used to allow for different epidemic trajectories in different areas. The most general representation would allow interactive autoregressive coefficients varying simultaneously by time and area. We also allow for area specific permanent effects $\varepsilon _{i}$ (and $e_{i}$) and space-time varying intercepts $\phi _{it}$ (and $f_{it}$).

The linear and log-linear representations, generalizing Eqs. (1) and (2) to area-time, become

3 $$\begin{aligned} \mu _{it}=\varepsilon _{i}+\phi _{it}+\alpha _{it}y_{i,t-1}+\beta _{it} \underset{}{\sum _{j}}w_{ij}y_{j,t-1}+\gamma _{it}\mu _{i,t-1}+\delta _{it} \underset{}{\sum _{j}}w_{ij}\mu _{j,t-1}, \end{aligned}$$

and

4 $$\begin{aligned}&\log (\mu _{it})=\nu _{it}=e_{i}+f_{it}+a_{it}\log (y_{i,t-1}+1)+b_{it} \underset{}{\sum _{j}}w_{ij}\log (y_{j,t-1}+1)+c_{it}\nu _{i,t-1}\nonumber \\&+d_{it} \underset{}{\sum _{j}}w_{ij}\nu _{j,t-1}. \end{aligned}$$

In Eq. (3), the $\{\varepsilon _{i},\phi _{it},\alpha _{it},\beta _{it},\gamma _{it},\delta _{it}\}$ are assumed positive under the identity link. Covariate effects can be included in the specifications for $\varepsilon _{i}$ and or $\phi _{it},$ and for $e_{i}$ and $f_{it},$ though arguably are more straightforwardly obtained under Eq. (4); see Fokianos and Tjøstheim (2011, page 564) regarding the time series case.

Assuming positive dependence on infection count lags is a reasonable prior assumption anyway, on subject grounds, as higher existing numbers of infected subjects typically generate more future infections. It is implausible that more infections in period *t* in area *i* generate less infections in period $t+1$. In Eq. (4), assuming positivity of the autoregressive coefficients $(a_{it},b_{it},c_{it},d_{it})$ is also a reasonable assumption, for the same reason. In practice, one may use log, or logit, links to space or space-time random effects. For example, a log-link involving fully interactive space-time structured random effects, $\psi _{it}$ (e.g. Lagazio et al. 2001, Eq. 4) on the lagged focus area infection counts is

5 $$\begin{aligned} log(\alpha _{it})=\alpha _{0}+\psi _{it}, \end{aligned}$$

with an intercept $\alpha _{0}$, and assuming the $\psi _{it}$ are constrained for identifiability (e.g. zero centred or corner constrained). Similar schemes can be applied to the other autoregressive coefficients.

However, including lags in latent means in Eqs. (3) and (4) will typically increase computational intensivity, and a more tractable model is based only on lags in observed infection counts or log transformed infection counts. Hence, the linear and log-linear specifications become

6 $$\begin{aligned} \mu _{it}=\varepsilon _{i}+\phi _{it}+\alpha _{it}y_{i,t-1}+\beta _{it} \underset{}{\sum _{j}}w_{ij}y_{j,t-1}, \end{aligned}$$

and

7 $$\begin{aligned} \log (\mu _{it})=e_{i}+f_{it}+a_{it}\log (y_{i,t-1}+1)+b_{it}\underset{}{ \sum _{j}}w_{ij}\log (y_{j,t-1}+1). \end{aligned}$$

Also area-time fully interactive specifications for autoregressive coefficients may be subject to overparameterisation (Regis et al. 2021, page 6), and one may propose reduced coefficient schemes. For example, for the lag term on $y_{i,t-1}$ in Eq. (6), one may take

8.1 $$\begin{aligned} log(\alpha _{it})=\alpha _{0}+\psi _{i} \end{aligned}$$

8.2 $$\begin{aligned} log(\alpha _{it})=\alpha _{0}+\psi _{t}. \end{aligned}$$

The option (8.1) is used in Paul and Held (2011), who assume $\psi _{i}$ are spatially structured random effects.

Here we investigate the gains—in the context of predicting future COVID-19 counts—of an autoregressive specification with separate area and time effects, for example in the linear model,

9 $$\begin{aligned} log(\alpha _{it})=\alpha _{0}+\psi _{1i}+\psi _{2t}, \end{aligned}$$

where $\psi _{1i}$ is a spatially structured conditional autoregressive or CAR effect (Besag et al. 1991), and $\psi _{2t}$ is a random walk in time. Both $\psi _{1i}$ and $\psi _{2t}$ are zero centred; for instance, such centering is automatically implemented in the BUGS car.normal function. This specification may provide greater adaptivity to rapidly changing infection counts in epidemic exponential and downturn phases, and avoids the heavy parameterisation of a fully interactive scheme.

### Remaining effects

For the permanent terms $\varepsilon _{i}$ and $e_{i}$, one might use iid or spatially correlated random effects $\kappa _{i}$ to represent enduring risk variations for infectious disease, in both endemic and epidemic phases. For example, taking iid effects, and with a positivity constraint,

10 $$\begin{aligned} \varepsilon _{i}=\exp (\kappa _{i}) \end{aligned}$$

where $\kappa _{i}\sim \mathcal {N}(\theta _{0},\sigma _{\kappa }^{2})$ are permanent effects across times. These terms might also include constant effects of covariates $X_{i}$. Thus for a single covariate

$$\begin{aligned} \varepsilon _{i}=\exp (\kappa _{i}) \end{aligned}$$

$$\begin{aligned} \kappa _{i}\sim \mathcal {N}(\theta _{0}+\theta _{1}X_{1i},\sigma _{\kappa }^{2}), \end{aligned}$$

where $\theta =(\theta _{0},\theta _{1})$ are regression parameters.

For the general time terms $\phi _{it}$ and $f_{it}$, various specifications are possible. These might include Fourier series representations for an infectious disease with clear seasonal fluctuations (Paul and Held 2011), or a second degree polynomial (in days) in a COVID-19 application (Giuliani et al. 2020). The latter scheme is proposed as adapting to the exponential growth in the upturn phase of the epidemic. There is no conclusive evidence so far that COVID-19 is seasonal. For example, the UK first COVID-19 wave peaked in the spring and early summer of 2020. Some studies argue that COVID will eventually become seasonal (e.g. Greene 2011). However, there will likely still be considerable variation between areas in timing of COVID infections.

Here, we use area-specific first-order random walks to (a) represent trends not fully captured by the autoregressive effects on infection lags and (b) be geographically adaptive. Thus in Eq (6), we have

11 $$\begin{aligned} \phi _{it}=\exp (\eta _{it}) \end{aligned}$$

where $\eta _{it}\thicksim \mathcal {N}(\eta _{i,t-1},\sigma _{\eta }^{2}).$ A corner constraint—setting selected parameter(s) to known values—is used for identifiability (Stegmueller 2014) and was less computationally intensive than centering samples at each iteration in the BUGS software. Thus, $\phi _{it}=\exp (\eta _{it}^{\prime }),$ where $\eta _{it}^{\prime }=\eta _{it}-\eta _{i1},$ which is equivalent to setting $\eta _{i1}=0$ (Lagazio et al. 2001, page 29).

The area specific effects $\eta _{it}$ will increase adaptivity. However, we also expect autoregressive coefficients including time effects, as in Eq. (9), to be adaptive to epidemic growth (and decay) phases. For example, in the growth phase with $y_{i,t+1}$ typically much exceeding $y_{it}$, the $\psi _{2t}$ in Eq. (9) will tend to be higher in order to better predict increasing counts $y_{i,t+1}$ in the next period.

The time varying terms $\phi _{it}$ and $f_{it}$ might also include time varying regression effects $\theta _{t}$, or impacts of time varying covariates, including lagged covariates (e.g. Lowe et al. 2021).

### Full model

In the case study analysis described below, we assume negative binomial sampling, with the linear model as in Eq (1) namely

$$\begin{aligned} \mu _{it}=\varepsilon _{i}+\phi _{it}+\alpha _{it}y_{i,t-1}+\beta _{it} \underset{}{\sum _{j}}w_{ij}y_{j,t-1}, \end{aligned}$$

and the log-linear, as in Eq (7), namely

$$\begin{aligned} \log (\mu _{it})=e_{i}+f_{it}+a_{it}\log (y_{i,t-1}+1)+b_{it}\underset{}{ \sum _{j}}w_{ij}\log (y_{j,t-1}+1). \end{aligned}$$

Initially, we take $w_{ij}$ to be first-order adjacency indicators: $h_{ij}=1$ for areas *i* and *j* adjacent, and $h_{ij}=0$ otherwise, with $w_{ij}=h_{ij}/\underset{}{\sum _{j}}h_{ij}.$ The autoregressive coefficients are taken as

12 $$\begin{aligned} log(\alpha _{it})=\alpha _{0}+\psi _{1i}+\psi _{2t} \end{aligned}$$

$$\begin{aligned} log(\beta _{it})=\beta _{0}+\psi _{3i}+\psi _{4t}, \end{aligned}$$

under the linear model, and

13 $$\begin{aligned} log(a_{it})=a_{0}+\psi _{5i}+\psi _{6t}, \end{aligned}$$

$$\begin{aligned} log(b_{it})=b_{0}+\psi _{7i}+\psi _{8t}, \end{aligned}$$

under the log-linear model. The parameters $\{\psi _{1i},\psi _{3i},\psi _{5i},\psi _{7i}\}$ are spatial CAR effects (Besag et al. 1991), and $\{\psi _{2t},\psi _{4t},\psi _{6t},\psi _{8t}\}$ are first-order random walks in time. The remaining effects are specified as

14 $$\begin{aligned} log(\varepsilon _{i})=\kappa _{1i}, \end{aligned}$$

$$\begin{aligned} log(\phi _{it})=\eta _{1it} \end{aligned}$$

$$\begin{aligned} \eta _{1it}\thicksim \mathcal {N}(\eta _{1i,t-1},\sigma _{\eta 1}^{2}), \end{aligned}$$

$$\begin{aligned} \kappa _{1i}\sim \mathcal {N}(\mu _{\kappa 1},\sigma _{\kappa 1}^{2}), \end{aligned}$$

in the linear model, and

15 $$\begin{aligned} e_{i}=\kappa _{2i} \end{aligned}$$

$$\begin{aligned} f_{it}=\eta _{2it}, \end{aligned}$$

$$\begin{aligned} \eta _{2it}\thicksim \mathcal {N}(\eta _{2i,t-1},\sigma _{\eta 2}^{2}), \end{aligned}$$

$$\begin{aligned} \kappa _{2i}\sim \mathcal {N}(\mu _{\kappa 2},\sigma _{\kappa 2}^{2}), \end{aligned}$$

in the log-linear model. The parameters $\{\alpha _{0},\beta _{0},a_{0},b_{0},\mu _{\kappa 1},\mu _{\kappa 2}\}$ are fixed effects.

Out-of-sample forecasts $\widetilde{y}_{i,T+s}$ for periods $T+1,T+2,...,etc.$ , are based on extrapolating $\psi _{2t},\psi _{4t},$ and $\eta _{1it}$ (or analogous log-linear effects) to provide means $\widetilde{\mu }_{i,T+s}$ (Sáfadi and Morettin 2003). Thus, one-step ahead predictions to $T+1$ in the linear model are

$$\begin{aligned} \psi _{2,T+1}\thicksim \mathcal {N}(\psi _{2T},\sigma _{\psi 2}^{2}), \end{aligned}$$

$$\begin{aligned} \psi _{4,T+1}\thicksim \mathcal {N}(\psi _{4T},\sigma _{\psi 4}^{2}), \end{aligned}$$

$$\begin{aligned} \eta _{1i,T+1}\thicksim \mathcal {N}(\eta _{1i,T},\sigma _{\eta 1}^{2}), \end{aligned}$$

and these are incorporated in Eq. (6) to provide $\mu _{i,T+1}$ from which forecast cases at $T+1$ can be sampled.

### Spatial weighting

There has been discussion on how to weight the contribution of neighbouring areas in the spatial lags, with proposals including a power law that has declining weights for second, third, etc., nearest neighbours (Cheng et al. 2016; Meyer and Held 2014). Here, we allow for an infection overspill effect from both first- and second-order neighbours in a sensitivity analysis.

Thus, first-order neighbours are assigned weights $h_{ij1}=1$ for adjacent areas, and $h_{ij1}=0$ otherwise; while second-order neighbours are assigned weights $0<\lambda <1,$ so that $h_{ij2}=\lambda$ for areas *i* and *j* which are second-order neighbours, and $h_{ij2}=0$ otherwise. Then,

16 $$\begin{aligned} w_{ij}=\frac{(h_{ij1}+h_{ij2})}{\underset{j}{\sum }(h_{ij1}+h_{ij2})}. \end{aligned}$$

### Space-time clusters

A range of methods have been proposed to assess space-time clustering (e.g. Chen et al. 2016; Mclafferty 2015). Here, we consider the LISA (Local Indicators of Spatial Association) indicator of spatial clustering in infection risk at one time point (Anselin 1995) and extend it to assess extended spatial clustering over various temporal windows—multiple successive time units (here these are successive weeks). A particular aim is to detect spatial clustering during the exponential ascent phase of the epidemic wave. Hence, one can assess where the epidemic phase, and its associated health care burden, is geographically concentrated.

Define predicted COVID case rates $r_{it}=\mu _{it}/P_{i}$, where $P_{i}$ are area populations. Predicted rates could also be defined for out-of-sample periods, with $\widetilde{r}_{it}=\widetilde{\mu }_{it}/P_{i},$ $t=T+1,T+2,...etc.,$ to predict future space-time risk patterns.

For a particular week define cluster indicators $J_{it}=1$ if own area rates $r_{it}$, and average rates in the locality $r_{it}^{L}=\underset{}{ \sum _{j\ne i}}w_{ij}r_{jt}/\underset{}{\sum _{j\ne i}}w_{ij},$ are both elevated. This is known as a high-high cluster in LISA terminology. If either or both of these conditions do not hold, then $J_{it}=0$.

Here, we define elevated rates as those more than 50% above the region wide or national rate—here the rate for the Greater South East, namely $$R_{t}= \underset{}{\sum _{i}}\mu _{it}/\underset{i}{\sum }P_{i}.$$ So $$J_{it}=1$$ if $$ J_{1it}=J_{2it}=1$$ where

17 $$\begin{aligned} J_{1it}=I(r_{it}>1.5R_{t}), \end{aligned}$$

$$J_{2it}=I(r_{it}^{L}>1.5R_{t}),$$

and where $I(C)=1$ if the comparison *C* is true, 0 otherwise.

Elevated rates through *D* successive weeks define a space-time cluster. So if $$D=5$$, a space-time cluster of length *D* would require $$ J_{it}=J_{i,t+1}=J_{i,t+2}=J_{i,t+3}=J_{i,t+4}=1$$. Using MCMC sampling one can obtain the probability that area *i* at week *t* defines a space-time cluster of length *D*.

### Estimation

Bayesian estimation uses the BUGS (Bayesian inference Using Gibbs Sampling) program (Lunn et al. 2009). Two chains of 20,000 iterations are taken, with inferences from the last 10,000, and convergence checks as in Brooks and Gelman (1998).

Gamma priors with shape one, and rate 0.001,  are adopted on inverse variance parameters and on the negative binomial overdispersion parameter $\Omega$, while normal $\mathcal {N}(0,100)$ priors are assumed on fixed effects $\{\alpha _{0},\beta _{0},a_{0},b_{0},\mu _{\kappa 1},\mu _{\kappa 2}\}.$ A beta(1,1) prior is assigned to $\lambda$ in the analysis including second-order neighbours.

## Case study

### Dataset and geographical setting

**Fig. 1:**
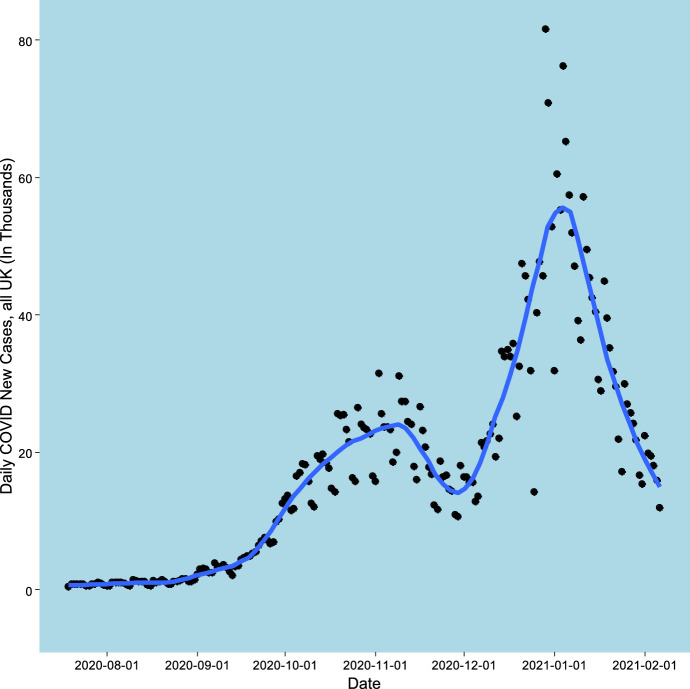
Daily New Cases across the UK. July 2020 to February 2021

The data for the study consist of weekly totals of new COVID cases in a subregion of the UK. The time span considered starts at the week 19-26 July 2020 (constituting week 1), with one analysis considering the subsequent 24 weeks, and another considering 29 weeks through to the week 31 January-6 February, 2021. In July 2020, new COVID cases across the entire UK averaged under 700 daily, whereas towards the end of 2020, there was a pronounced increase, with some days reaching over 75 thousand; however, in early 2021, there was a tailing off in new cases. See Fig. 1 for daily UK data, which includes a loess smooth. The epidemic ascent phase is irregular, with an early lesser peak in October and early November 2020, a slight tailing off in new cases in early December 2020, then a major increase in late December and January 2021.

**Fig. 2:**
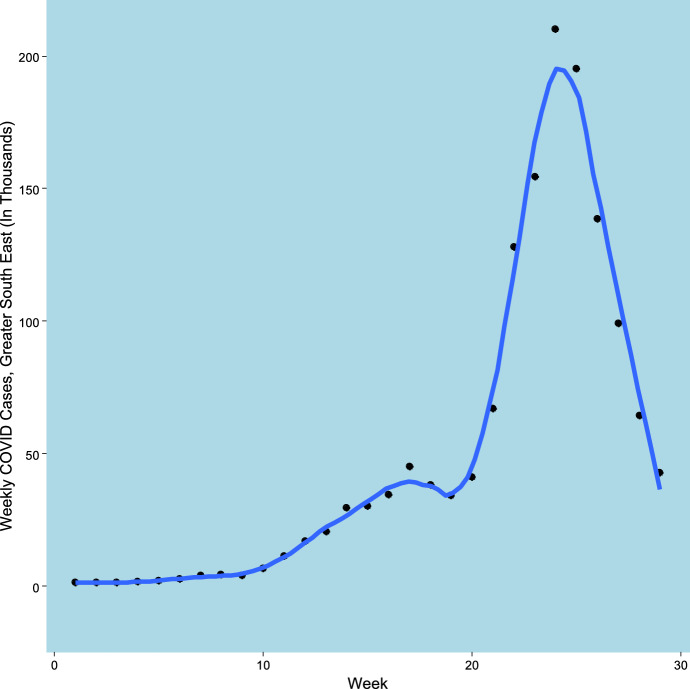
Weekly Totals of New COVID-19 Cases, Greater South East, July 2020 to February 2021

The analysis here considers part of England, namely three standard regions (London, South East, East) combined to give a broad region, here termed the Greater South East (GrSE for short), with a population of 24.4 million. Figure 2 shows weekly totals of new cases in this region. Starting at under 1,500 weekly, they rose to over 200,000 at the epidemic peak (on week 24) but then fell back sharply. As for the entire UK, there is a minor peak at week 17, preceding the main epidemic wave. There are $N=144$ areas in the region, administrative areas called local authorities.

This part of England contains the epicentre of a localized cluster associated with a new variant (the Kent variant, or B.1.1.7 variant) (Challen et al. 2021). The surge in new cases associated with this cluster was the precursor to the larger national UK-wide escalation of cases. The outbreak of the new variant was concentrated in areas to the east of London (in Kent and Essex counties) and in the North East of London itself.

### Model evaluations

As a first evaluation of alternative model forms, we make out-of-sample predictions for cases at weeks 24 and 29 across the Greater South East. The forecasts are based on training data for weeks 1-23, and weeks 1-28, respectively (so $T=23$ and $T=28$ respectively). Week 24 followed the ascent phase, when new cases of infection were sharply increasing, and in fact infections peaked in week 24. Week 29 was in a phase of sharp decline in new cases.

In a first analysis, a comparison between two different autoregressive formulations (*M*1 and *M*2) is made. Both specifications condition on the first week ($t=1$). Both specifications also assume a linear model, as in Eqs (6) and (12), namely

18 $$\begin{aligned} \mu _{it}=\varepsilon _{i}+\phi _{it}+\alpha _{it}y_{i,t-1}+\beta _{it} \underset{}{\sum _{j}}w_{ij}y_{j,t-1},\qquad \qquad t=2,...,T, \end{aligned}$$

$$log(\varepsilon _{i})=\kappa _{1i},$$

$$log(\phi _{it})=\eta _{1it},$$

$$\begin{aligned} \eta _{1it}\thicksim \mathcal {N}(\eta _{1i,t-1},\sigma _{\eta 1}^{2}), \end{aligned}$$

$$\begin{aligned} \kappa _{1i}\sim \mathcal {N}(\mu _{\kappa 1},\sigma _{\kappa 1}^{2}). \end{aligned}$$

In the first specification (*M*1), the autoregressive coefficients $\alpha _{it}$ and $\beta _{it}$ are taken as spatially, but not temporally, varying:

19 $$\begin{aligned} log(\alpha _{it})=\alpha _{0}+\psi _{1i}, \end{aligned}$$

$$log(\beta _{it})=\beta _{0}+\psi _{3i},$$

In the second (*M*2), the autoregressive coefficients are taken as both space and time varying

20 $$\begin{aligned} log(\alpha _{it})=\alpha _{0}+\psi _{1i}+\psi _{2t}, \end{aligned}$$

$$log(\beta _{it})=\beta _{0}+\psi _{3i}+\psi _{4t}.$$

The parameters $\{\psi _{1i},\psi _{3i}\}$ are CAR effects (Besag et al. 1991), with $h_{ij}=1$ for adjacent areas ($h_{ij}=0$ otherwise), while $\{\psi _{2t},\psi _{4t}\}$ are first-order random walks in time.

One-step ahead out-of-sample forecasts $\widetilde{y}_{i,T+1}$ for week $T+1$ (either week 24 or week 29) are based on extrapolating $\psi _{2t},\psi _{4t},$ and $\eta _{1it}$ to week $T+1$.

Two subsequent analyses are made. In the first, we compare the best performing from *M*1 and *M*2 with its log-linear equivalent (*M*3). In the second analysis, we allow the spatial interaction weights $w_{ij}$ to include both first- and second-order neighbours—this defines model *M*4. Both these analyses are for the case when $T=23,$ and out-of-sample predictions are to week 24.

### Assessing performance

Out-of-sample predictive performance is based on whether the 95% credible interval for predicted new cases $\widetilde{y}_{\bullet ,T+1}$ (summing across 144 areas in the GrSE) in week $T+1$ contains the actual number of new cases $y_{\bullet ,T+1}$. An indicator of this is the posterior probability

21 $$\begin{aligned} \zeta =Pr(\widetilde{y}_{\bullet ,T+1}>y_{\bullet ,T+1}|y) \end{aligned}$$

that one-step ahead predicted cases exceed actual new cases. Tail probabilities (e.g. under 0.1 or over 0.9) represent under or over-prediction of actual cases. These probabilities can be obtained for individual areas, namely

22 $$\begin{aligned} \zeta _{i}=Pr(\widetilde{y}_{i,T+1}>y_{i,T+1}|y). \end{aligned}$$

Also considered is the ranked probability score, with abbreviation $RPS_{T+1}$ Czado et al. (2009), which measures the accuracy of forecasts (in matching actual outcomes) when expressed as probability distributions. In a Bayesian context, the latter will be sampled values from posterior predictive densities for the outcome, $p(\widetilde{y}|y).$ For area *i*, the ranked probability score is obtained by monitoring

$$\begin{aligned} \left| \widetilde{y}_{i,T+1}-y_{i,T+1}\right| +\left| \widetilde{y}_{i,T+1}-\overset{\thickapprox }{y}_{i,T+1}\right| \end{aligned}$$

where $\overset{\thickapprox }{y}_{i,T+1}$ is an independent draw for the posterior predictive density. The second term is a penalty for uncertainty, which increases as does predictive variance. Lower $RPS_{T+1}$ values represent better fit.

To assess fit for the observed (training) data, we obtain the widely applicable information criterion (*WAIC*) (Watanabe 2010), and also *RPS* scores for one-step ahead predictions, based on infections in the previous week. The *RPS* scores can be aggregated over areas for separate weeks, $$ RPS_{t}$$ $$(t=2,...,T),$$ to show where particular models are better or worse fitting.

## Results

### Predictive performance of space-time autoregression model

Table 1 compares the out-of-sample performance of models *M*1 and *M*2 for weeks 24 and 29, based, respectively, on training data for weeks 1-23 and 1-28. Table 2 compares model fit for the training data analysis, as well as predictive performance for one-step ahead predictions within the sample.

**Table 1:** Out-of-Sample Predictions, Models *M1* and *M2* Compared

	*M1*		*M2*	
	Spatially Varying Autoregression, Linear Model (*M1*)		Space-Time Varying Autoregression, Linear Model (*M2*)	
	Week 24	Week 29	Week 24	Week 29
Actual Cases	210,099	42,987	210,099	42,987
Mean Predicted Cases	195,533	72,960	228,498	49,080
Median Predicted Cases	195,338	72,870	209,027	44,930
Predicted Cases (2.5%)	181,775	77,340	105,328	15,860
Predicted Cases (97.5%)	210,807	99,590	449,227	78,560
Prob(Prediction Exceeds Actual), $\zeta$	0.03	1	0.49	0.55
Number of Areas with $\zeta _{i}$ > 0.9 or < 0.1	25	61	8	1
Ranked Probability Score (Mean)	131,700	59,610	133,559	27,850
Ranked Probability Score (Median)	131,300	59,410	104,038	21,380

Notes: $\zeta$ is posterior probability that predicted cases (first out of sample period) exceed actual cases; $\zeta _{i}$ are area-specific posterior probabilities that predicted cases (first out of sample period) exceed actual cases

It can be seen from Table 1 that a model including time effects in the autoregressions on previous cases leads to improved out-of-sample predictions. The credible intervals under *M*2 for predicted new cases in weeks 24 and 29 comfortably include the actual total GrSE cases; though the *M*2 estimates of total cases are less precise and show some skew (posterior means exceeding medians). The mean RPS score under *M*2 also shows the effects of skewness, especially for the forecast to $T+1=24$; the median values favour *M*2.

**Table 2:** In-Sample (Training Data) Fit, and One-Step Ahead In-Sample Predictions, Models *M1* and *M2*

	*M1*		*M2*	
	Space Varying Autoregression, Linear Model		Space-Time Varying Autoregression, Linear Model	
Training Data Period	Weeks 1-23	Weeks 1-28	Weeks 1-23	Weeks 1-28
WAIC	29,905	40,231	28,169	37,762
RPS, Total	475,698	1,123,105	311,565	559,265
RPS, week 2	1605	1548	1092	1076
RPS, week 3	1625	1587	1147	1122
RPS, week 4	1697	1693	1346	1311
RPS, week 5	2056	2053	1562	1514
RPS, week 6	2469	2459	1803	1734
RPS, week 7	2993	3052	2411	2315
RPS, week 8	3884	3837	2621	2504
RPS, week 9	4672	4539	2503	2362
RPS, week 10	4734	4926	3733	3576
RPS, week 11	7353	7621	6093	5764
RPS, week 12	10,660	11,050	8192	7676
RPS, week 13	14,200	14,320	9345	8721
RPS, week 14	17,920	18,570	13,483	12,450
RPS, week 15	24,560	23,770	14,035	12,980
RPS, week 16	24,300	24,170	15,938	14,810
RPS, week 17	28,050	28,510	19,510	18,120
RPS, week 18	39,220	36,690	17,098	15,880
RPS, week 19	33,150	31,440	14,996	13,990
RPS, week 20	28,200	28,420	17,962	16,690
RPS, week 21	39,920	42,100	29,046	26,940
RPS, week 22	77,630	81,870	54,341	50,580
RPS, week 23	104,800	100,900	73,309	64,270
RPS, week 24		128,400		84,890
RPS, week 25		156,100		73,630
RPS, week 26		161,600		52,250
RPS, week 27		114,000		37,370
RPS, week 28		87,880		24,740

Notes: WAIC, widely applicable information criterion; RPS, Ranked Probability Score

The probabilities $\zeta$ in Eq. (21) indicate that model *M*1 underpredicts new cases at week 24; this week was in fact the peak of the epidemic, following weeks when actual cases were rapidly increasing. By contrast, in the downturn phase, at week 29, model *M*1 overpredicts new cases. Area specific probabilities $\zeta _{i}$, as in Eq. (22), show higher totals of local authority areas with cases under or overpredicted under *M*1, especially in the downturn phase.

Table 2 shows that model *M*2 has a lower in-sample WAIC than model *M*1 in both training data analyses. One-step ahead predictions within the observed data periods also favour *M*2. For example, the total *RPS* for *M*1, accumulated over weeks 1-28, is around twice that for *M*2 (1.12 million vs 559 thousand). Some weeks show greater discrepancies between the models.

Table 3 compares the two models against information on changing infection totals (weekly totals across GrSE) for the analysis of weeks 1-28. Comparing $RPS_{t}$ between models *M*1 and *M*2 (first three columns of Table 3) shows that model *M*1 has problematic fit in the irregular ascent phase (weeks 16-19 when cases rise then fall back again), and also, more markedly, in the epidemic descent phase (weeks 26 onwards), when the $RPS_{t}$ under *M*1 is more than three times that of *M*2.

**Table 3:** Comparative Fit by Week, Models *M1* and *M2*, Weeks 1-28

	*M1*	*M2*	RPS Ratio, *M1* vs *M2*	Total Cases, Greater South East	$\psi _{2t}$ in *M2* (Posterior Means)
RPS, Week 2	1548	1076	1.44	1312	– 0.96
RPS, Week 3	1587	1122	1.41	1407	– 1.40
RPS, Week 4	1693	1312	1.29	1755	– 1.55
RPS, Week 5	2053	1515	1.36	2161	– 1.69
RPS, Week 6	2459	1734	1.42	2641	– 1.33
RPS, Week 7	3052	2315	1.32	3972	– 0.90
RPS, Week 8	3837	2506	1.53	4458	– 0.79
RPS, Week 9	4539	2362	1.92	4030	– 0.99
RPS, Week 10	4926	3578	1.38	6846	– 0.34
RPS, Week 11	7621	5767	1.32	11,365	0.60
RPS, Week 12	11,050	7679	1.44	17,034	0.70
RPS, Week 13	14,320	8727	1.64	20,523	0.56
RPS, Week 14	18,570	12,460	1.49	29,633	0.84
RPS, Week 15	23,770	12,990	1.83	30,263	0.52
RPS, Week 16	24,170	14,820	1.63	34,546	0.53
RPS, Week 17	28,510	18,120	1.57	45,007	0.68
RPS, Week 18	36,690	15,890	2.31	38,227	0.09
RPS, Week 19	31,440	14,000	2.25	34,345	0.16
RPS, Week 20	28,420	16,710	1.70	41,090	0.50
RPS, Week 21	42,100	26,950	1.56	67,090	0.82
RPS, Week 22	81,870	50,620	1.62	127,905	1.09
RPS, Week 23	100,900	64,310	1.57	154,518	0.93
RPS, Week 24	128,400	84,950	1.51	210,099	1.11
RPS, Week 25	156,100	73,660	2.12	195,055	0.61
RPS, Week 26	161,600	52,290	3.09	138,553	0.37
RPS, Week 27	114,000	37,390	3.05	99,205	0.33
RPS, Week 28	87,880	24,750	3.55	64,354	0.24

*RPS* Ranked Probability Score; $\psi _{2t}$ are Time Dependence Effects in Space-Time Autoregression Parameters

**Fig. 3:**
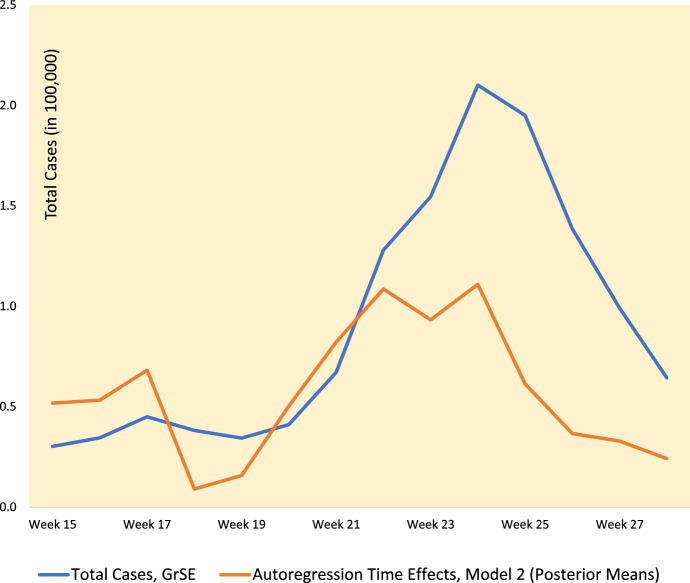
Autoregressive Time Parameters and Total Infections (in units of 100,000)

The last two columns of Table 3 and Fig. 3 show how the $\psi _{2t}$ in model *M*2 adapt to the minor early peak at week 17, and then to sharply increasing cases in the exponential epidemic phase. They then decrease in line with the epidemic downturn.

### Evaluating other model options

Table 4 compares linear and log-linear specifications (denoted *M*2 and *M*3) with space-time autoregressive effects, where the log-linear model *M*3 is defined by Eqs. (7), (13) and (15). This comparison is for weeks 1-23 as training data, and prediction ahead to week 24. For *M*3, we find a slight deterioration in fit to the training data and also a slight deterioration in out-of-sample prediction—though the latter is still satisfactory. However, skewness in the posterior density of $\widetilde{y}_{\bullet ,T+1}$ is increased in *M*3 as against *M*2.

**Table 4:** Out-of-Sample Predictions and In-sample Fit, Models *M2*, *M3* and *M4* Compared, $$T = $$ 23

	*M2*	*M3*	*M4*
	Space-Time Varying Autoregression, Linear Model. First-Order Neighbours Only	Space-Time Varying Autoregression, Log-Linear Model. First-Order Neighbours Only	Space-Time Varying Autoregression, Linear Model. First- and Second-Order Neighbours
	$$T = $$ 23	$$T = $$ 23	$$T = $$ 23
*Out-of- Sample Predictions (to week 24)*			
Actual Cases	210,099	210,099	210,099
Median Predicted Cases	209,027	193,145	211,272
Predicted Cases (2.5%)	105,328	52,751	94,353
Predicted Cases (97.5%)	449,227	1,257,550	581,146
Prob(Prediction Exceeds Actual), $\zeta$	0.49	0.46	0.51
Number of Areas *i* with $\zeta _{i}$ > 0.9 or < 0.1.	8	0	3
RPS (Median)	104,038	142,418	114,208
*In-sample Fit*			
WAIC	28,169	28,907	28,814
RPS (Total)	311,565	355,956	332,257
RPS, week 2	1092	1188	1117
RPS, week 3	1147	1248	1176
RPS, week 4	1346	1425	1390
RPS, week 5	1562	1674	1610
RPS, week 6	1803	2002	1878
RPS, week 7	2411	2666	2534
RPS, week 8	2621	2860	2729
RPS, week 9	2503	2761	2617
RPS, week 10	3733	4064	3917
RPS, week 11	6093	6831	6330
RPS, week 12	8192	9434	8793
RPS, week 13	9345	10,680	10,002
RPS, week 14	13,483	15,468	14,474
RPS, week 15	14,035	15,818	15,097
RPS, week 16	15,938	18,204	17,125
RPS, week 17	19,510	22,919	21,058
RPS, week 18	17,098	19,482	18,290
RPS, week 19	14,996	17,433	15,953
RPS, week 20	17,962	21,122	19,392
RPS, week 21	29,046	34,314	31,353
RPS, week 22	54,341	64,142	58,238
RPS, week 23	73,309	80,223	77,187

*WAIC* Widely applicable information criterion, *RPS* Ranked probability score; $\zeta$ is posterior probability that predicted cases (first out of sample period) exceed actual cases; $\zeta _{i}$ are area-specific posterior probabilities that predicted cases (first out of sample period) exceed actual cases

Another version of the linear model is also considered (as *M*4), with spatial weights $w_{ij}$ including second-order as well as first-order neighbours—as per Eq. (16). For model *M*4, we find no gain in fit over model *M*2 using first-order neighbours only. The out-of-sample prediction is satisfactory though, with no evidence of under or overprediction of cases in week $T+1$. The posterior median estimate of $\widetilde{y}_{.,T+1},$ namely new cases in week $T+1$ across the greater South East, is 211,272 compared to the actual total of 210,099. The $\lambda$ parameter has mean 0.76 with 95% credible interval (0.40, 0.99).

### Detecting significant space-time clusters

Space-time clustering in infectious disease outbreaks is important in identifying the epicentre(s) of an outbreak. Space-time cluster prediction, for example to assess continued excess spatial clustering in future periods, is important in prioritizing interventions.

The “Kent variant” of COVID-19 (code B117), also known as the “English variant”, emerged in late 2020 in specific parts of England, namely areas to the East and South East of London. The observed data suggest a localized surge of COVID-19 cases in November 2020 in these locations, which preceded the generalized national second wave epidemic peaking in late December of 2020 and early January of 2021. In terms of the weeks considered in the present study, we would expect significant space-time clustering in weeks 17-22, namely November 2020 and early December 2020.

We obtain—under model *M*2—area specific probabilities of *D* successive periods with excess incidence in both focus areas and their localities. Excess incidence is taken as more than 50% above the average (modelled) rate for the entire region, namely the Greater South East. Assuming $D=5$, then for a single MCMC iteration ($s=1,...,S$), one requires for area *i* to be a space-time cluster of length 5 that $J_{it}^{(s)}=J_{i,t+1}^{(s)}=J_{i,t+2}^{(s)}=J_{i,t+3}^{(s)}=J_{i,t+4}^{(s)}=1.$ One then obtains estimated posterior probabilities of such a sequence occurring, by accumulating over MCMC iterations.

**Fig. 4:**
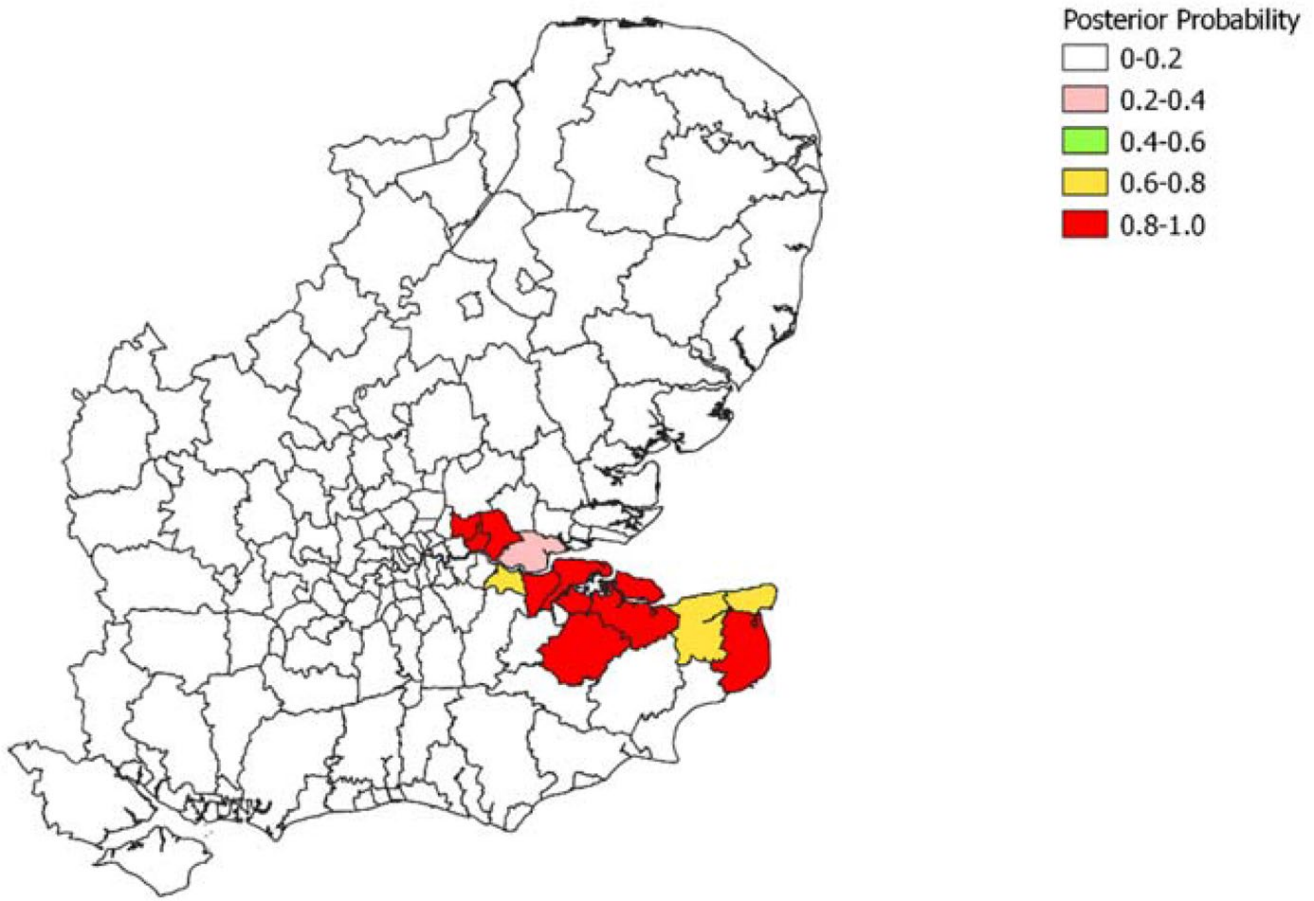
Posterior Probabilities of Space-Time Cluster of Length Four Weeks During Epidemic Ascent Phase, Local Authorities, Greater South East of England

**Fig. 5:**
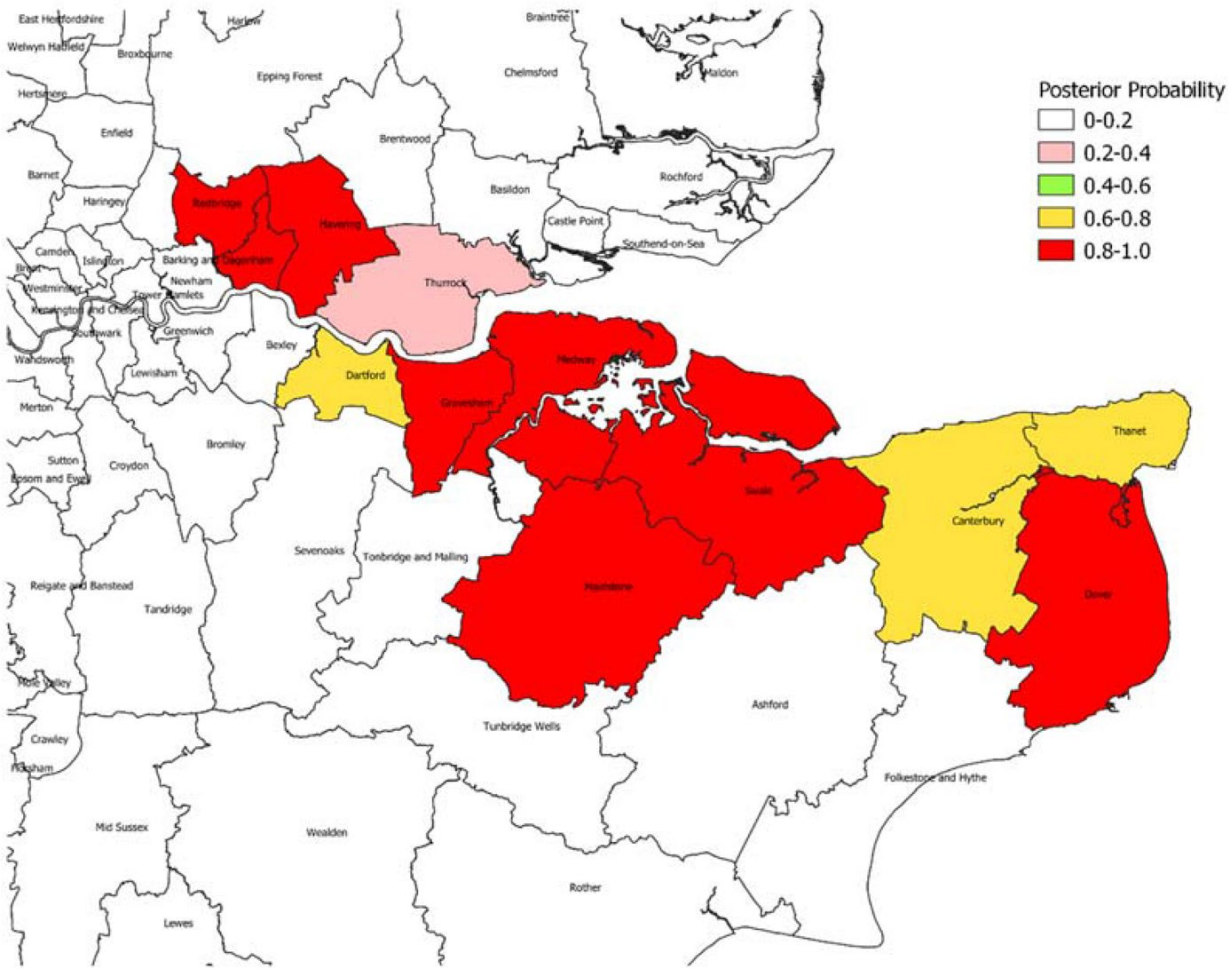
Posterior Probabilities of Space-Time Cluster of Length Four Weeks. Detailed Focus

Focussing on weeks 17-22, we find only one area with a posterior probability exceeding 0.9 of being centre of a persistent space-time cluster of length 5 weeks. However, considering persistent clusters of length $D=4$ weeks, there are seven areas with probabilities over 0.9, and eight areas with probabilities over 0.8. Figure 4 shows the estimated probabilities for $D=4$ across the Greater South East of England, with a sharp delineation apparent between the “Kent variant” epicentre, and other areas. Figure 5 shows in closer detail the areas in the epicentre. The Swale local authority, with a posterior probability of one, was among the Kent local authorities first affected by the new variant (Reuters 2021).

Of interest also are forecasts of clustering status. We consider training data for the first $T=23$ weeks to make one-step ahead predictions of clustering in week 24. So cluster indicators $J_{i,T+1}=1$ if own area rates $\widetilde{r}_{i,T+1}=\widetilde{\mu }_{i,T+1}/P_{i}$, and average rates in the locality $\widetilde{r}_{i,T+1}^{L}=\underset{}{\sum _{j\ne i}}w_{ij} \widetilde{r}_{j,T+1}/\underset{}{\sum _{j\ne i}}w_{ij},$ are both elevated as compared to the region wide rates, $\widetilde{r}_{T+1}=\underset{}{ \sum _{i}}\widetilde{\mu }_{i,T+1}/\underset{}{\sum _{i}}P_{i}.$ Rates more than 50% above the region wide rate are considered elevated.

In such short-term forecasting, one may compare predicted future clustering with “actual” clustering defined by observed disease counts. Thus, actual rates for area *i* are $y_{i,T+1}/P_{i}$, with corresponding locality averages and region-wide rates; these are reliable point estimators for large disease counts. In fact, seven of the 144 areas are identified as actual cluster centres at week 24, the epidemic peak. Predicted and actual cluster status are compared using a $2\times 2$ table accumulating correct classifications along the diagonal (areas where both actual and predicted cluster status are the same). We can then assess sensitivity, the proportion of actual high-high cluster centres correctly identified, and specificity, the proportion of non-cluster areas correctly identified.

Under model *M*2, we obtain posterior mean sensitivity (with 95% credible interval) of 0.93 (0.43,1.0), and posterior mean specificity of 0.965 (0.95,0.985). The model prediction is for slightly higher numbers of cluster centres than is actually the case (false positives, with posterior mean 4.8), and this reduces specificity. False negatives are infrequent, with posterior mean 0.5. Using the relationship accuracy = (sensitivity)(prevalence) + (specificity)(1 - prevalence), where the prevalence of high-high clustering is 7/144, one obtains an accuracy of around 0.964.

### Covariate effects

There have been many studies on socio-demographic and environmental risk factors for COVID outcomes. Both incidence and mortality have been linked to area deprivation, urbanicity, poor air quality, and nursing home location (as area risk factors), and non-white ethnicity, and existing medical conditions (as individual risk factors). Impacts of such risk factors were clearly observable in the UK first wave of the COVID pandemic, concentrated in March to May of 2020 (Public Health England 2020; O’Dowd 2020; Quinio 2021; Dutton 2020).

The second UK wave is distinct from the first, in being strongly linked to the emergence of a new virus strain, and by the form of geographic clustering associated with the new strain (see section 5.3), namely a concentration in non-metropolitan areas in the south east of England, areas with relatively low concentrations of ethnic groups and area deprivation. This may tend to attenuate or distort the effect of area predictors $X_{i}$, so that although their inclusion may improve fit and predictions, the substantive rationale for including them—as disease risk factors per se—is in doubt.

To illustrate this potential for distortion, we estimate a time-varying effect of rurality on COVID infection rates. Rurality in each local authority (LA) is measured by the proportion of micro-areas (lower super output areas) within each LA that are classified as rural towns or villages (Office of National Statistics 2013, Table 1b). One would expect rural areas, with lower population densities, to have lower infection and mortality rates (Lai et al. 2020). Matheson et al. (2020) attribute excess urban mortality (in the UK first COVID wave) to higher population density and association, more people-facing occupations in cities, and greater home overcrowding.

**Fig. 6:**
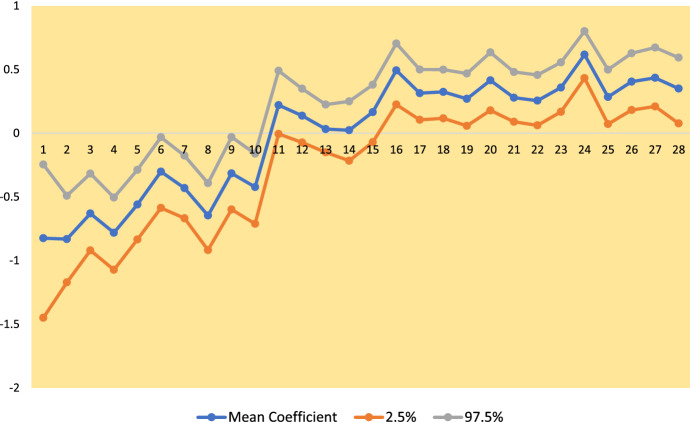
Time Varying Effect of Rurality, ${\theta}_{t}$

To establish its role for the second wave data, a regression analysis (with $$ T=28$$ weeks) is carried out with a time varying effect of rurality ($$X_{i}$$ ), using the log-linear model. Thus, Eq (6) is extended to include an additive term $$\theta _{t}X_{i},$$ where $$\theta _{t}$$ is a first-order random walk, with prior $$\theta _{t}\sim \mathcal {N}(\theta _{t-1},\sigma _{\theta }^{2}).$$ We find an irregular effect on infection rates, with $$ \theta _{t}$$ significantly negative in the early weeks of the study period, significantly positive in some later weeks, and often non-significant, with 95% intervals including zero—see Figure 6.

## Discussion and future research

Forecasts of future infectious disease incidence, especially with spatial disaggregation, are important for policy purposes in epidemic situations. There are benefits in a longitudinal model form which borrows strength over areas since incidence levels tend to be spatially clustered—an example being the geographically concentrated COVID-19 outbreak associated with the “Kent variant” in the UK. Subsequent epidemic diffusion will also be influenced by spatial proximity. Hence, several models in the literature allow spatially varying autoregressive effects, and spatially varying dependence on infection levels in nearby areas.

However, temporal adaptivity and forecasting performance may be improved by allowing for time variation in the epidemic path, for example through space-time autoregressive dependencies. An econometric perspective on autoregressive dependence allowing for both heterogeneity over units and over time is provided by Regis et al. (2021), though they suggest (Regis et al. 2021, p. 6)—from a classical estimation perspective—that a full unit-time random effect structure may be overparameterized.

A full spatio-temporal structure may be applied when longitudinal data cover a relatively short period and made identifiable subject to appropriate constraints. Thus, (Watson et al. 2017)—using a Bayesian perspective—consider area data on Lyme disease over $T=5$ years. They use the first four years to predict the last, using a full spatio-temporal autoregressive scheme allowing both spatial and temporal correlation.

However, over a longer set of time points, there would be a heavy parameterisation in a fully interactive scheme. In the present application, fully interactive autoregressive effects as in Eq. (5), and other space-time parameters as in Eq. (11), would involve 3*NT* unknown random effects (i.e. three times the number of data points). By contrast, the newly proposed space-time model—for example, in Eq. (9)—involves considerably fewer, $NT+2(N+T)$, random effects. A fully interactive specification would also limit the form of the time dependence in autoregression that can be considered; for example, a low order polynomial in time might be used for $\{\psi _{2t},\psi _{4t}\}$ in Eq. (12), instead of a random walk in time. Finally, with the separate space and time effects, as in Eq. (9), their distinct contribution to improved fit and forecasts can be assessed, and interpretability is straightforward.

In the present study, over a longitudinal series of nearly 30 time points, the parsimonious space-time autoregressive representation provides improved one-step ahead forecasts as compared to a model allowing spatially varying autoregressive dependence only. The latter model is shown to underpredict new cases at in the ascent phase of the epidemic (in November and early December 2020 for the UK second COVID-19 wave), when actual cases were rapidly increasing. By contrast, in the downturn phase, the model with only spatial variation in autoregressive effects provides an overprediction of new cases.

Other substantive features of infectious epidemics have been be investigated, such as the location of prolonged space-time clusters. In the Greater South East of England, there is a clearly demarcated epicentre for the outbreak in the epidemic ascent phase (see Fig. 4).

Drawing on the time series literature on random coefficient autoregression, we have set out alternative linear and log-linear specifications applicable to the area-time situation. For the particular infectious disease data concerned, the linear model had a better fit, but further research on similar forms of data (including longitudinal area data on chronic as well as infectious disease, and indeed any form of longitudinal area count data) is indicated to establish the comparative strengths of the linear and log-linear forms. The above analysis has not considered the full scope of possible autoregressive dependence—including lags on latent means for both the focus area and its locality—as in Eqs. (3) and (4). Such a model was not tractable in the software used here. Extensions may also be envisaged to higher order lags, such as spatio-temporal AR1 (lag 1) and AR2 (lag 2) dependence for both the focus area and its surrounding locality in Eqs. (6) and (7).

Given that the COVID pandemic has typically involved multiple waves, one might also be interested in longitudinal modelling over two or more waves, for instance to compare area-specific infection rates at epidemic peaks. The method used here is more easily applied to multiwave data than one involving area specific phenomenological models (e.g. logistic, Richards) which would necessitate using latent switching parameters between waves.

Another generalisation is to related outcomes such as mortality and hospitalisations. This could involve generalisations of the linear and log-linear count regression specifications—such as Eqs. (6) and (7)—to include borrowing strength over space, time and outcomes. This would be combined with multiple outcome count regression (Poisson or negative binomial). Alternatively, conditioning on modelled infections, one could model case fatality and hospitalisation as binomial responses .

## Declarations

### Code and Data availability

BUGS code and data for replication purposes are available at https://figshare.com/articles/software/SPATIOTEMP_REPLICATION_VERSION_txt/16628167
